# Osteo-NeT: An Automated System for Predicting Knee Osteoarthritis from X-ray Images Using Transfer-Learning-Based Neural Networks Approach

**DOI:** 10.3390/healthcare11091206

**Published:** 2023-04-23

**Authors:** Hassan A. Alshamrani, Mamoon Rashid, Sultan S. Alshamrani, Ali H. D. Alshehri

**Affiliations:** 1 Radiological Sciences Department, College of Applied Medical Sciences, Najran University, Najran 11001, Saudi Arabia; 2 Department of Computer Engineering, Faculty of Science and Technology, Vishwakarma University, Pune 411048, India; 3 Research Center of Excellence for Health Informatics, Vishwakarma University, Pune 411048, India; 4 Department of Information Technology, College of Computers and Information Technology, Taif University, Taif 21944, Saudi Arabia

**Keywords:** osteoarthritis, transfer learning, X-ray, CNN, VGG-16, ResNeT-50, automated system

## Abstract

Knee osteoarthritis is a challenging problem affecting many adults around the world. There are currently no medications that cure knee osteoarthritis. The only way to control the progression of knee osteoarthritis is early detection. Currently, X-ray imaging is a central technique used for the prediction of osteoarthritis. However, the manual X-ray technique is prone to errors due to the lack of expertise of radiologists. Recent studies have described the use of automated systems based on machine learning for the effective prediction of osteoarthritis from X-ray images. However, most of these techniques still need to achieve higher predictive accuracy to detect osteoarthritis at an early stage. This paper suggests a method with higher predictive accuracy that can be employed in the real world for the early detection of knee osteoarthritis. In this paper, we suggest the use of transfer learning models based on sequential convolutional neural networks (CNNs), Visual Geometry Group 16 (VGG-16), and Residual Neural Network 50 (ResNet-50) for the early detection of osteoarthritis from knee X-ray images. In our analysis, we found that all the suggested models achieved a higher level of predictive accuracy, greater than 90%, in detecting osteoarthritis. However, the best-performing model was the pretrained VGG-16 model, which achieved a training accuracy of 99% and a testing accuracy of 92%.

## 1. Introduction

Knee articular cartilage degeneration, also known as osteoarthritis (OA), is a type of joint disease that develops due to the breakdown of the joint cartilage, affecting 1 out of 7 adults in the world [1]. It is one of the major causes of disability in the world. The most common symptoms of this disease are pain and stiffness in the joints, which progress slowly over time. The most affected joints are the knee and hip joints and the joints of the fingers [2]. OA is caused by a large amount of mechanical stress on the joints and a low-grade inflammatory process. This progresses as the cartilage is lost and the bone underlying it becomes affected [3]. Generally, OA is diagnosed based on symptoms, along with medical imaging techniques. Recent studies [4,5,6] have concluded that a single clinical examination is not reliable for the diagnosis of OA, and knee arthroscopy is often performed to get a clear idea regarding the progression of OA. These studies have also explained that knee arthroscopy is an invasive process in which small cameras are inserted through small incisions for a direct overview of the knee cartilage. Furthermore, these studies have also shown that non-invasive techniques such as CT scans and MRIs are also used, but they are not as accurate as knee arthroscopy because radiologists may not correctly diagnose the condition due to a lack of expertise. This challenge prompted us to look for automated diagnostic systems. At present, there is no cure for OA, but there are certain factors, including biological and environmental risk factors, modifiable and non-modifiable, that are responsible for the development of OA [7]. OA belongs to the class of big data problems in terms of data size and complexity, and various machine learning techniques have been suggested in recent times for developing automated diagnostic systems for OA. Studies such as [8,9] have suggested the use of regression models for automated OA diagnostic systems. However, the authors of [7] reported that these models are not able to handle large, heterogeneous datasets to identify relationships between environmental and biological risk factors or analyze disease progression in a patient.

Recent advancements in machine learning in healthcare have led us to accelerate and improve the diagnostic process of various life-threatening diseases, including rheumatology and OA [10,11]. In this paper, we suggest an advanced automated OA diagnostic system based on neural networks and transfer learning. Using transfer learning will resolve the challenges faced by single trained machine learning models. Using transfer learning (TL) will help in reducing the computational time required for training the model, and this will also reduce the need for large high-quality datasets for developing a single diagnostic model, as the TL model will be pretrained. In rheumatology, this technique will help doctors improve their knowledge of the patient’s disease trajectory, as well as make care-related decisions and response-to-disease predictions based on immunological signals [10]. This paper contributes to the field of rheumatology as follows:

- This paper suggests and analyzes automated diagnostic and classification methods for knee osteoarthritis, along with a comparison with single trained machine learning diagnostic methodologies.
- This paper analyzes the effectiveness of a neural network in the classification of knee osteoarthritis through relevant performance judgment metrics.
- This paper also investigates the various data augmentation techniques used to train a model with high predictive accuracy.

The overall structure of the paper is as follows: Section 2 explains the related previous works in the field of automated diagnostic methods; Section 3 explains the suggested methodology, followed by an explanation of the results in Section 4. Finally, the paper is concluded in Section 5.

## 2. Previous Works

Osteoarthritis affects more than 3% of the population globally and is a major cause of disability in adults [12]. The diagnosis of OA is currently made through arthroscopy. Making an effective diagnosis is a complex process and requires a longer training time for a large dataset [10,12]; thus, automated systems based on machine learning techniques have been proven to be more efficient in the diagnosis and classification of OA from medical images. Various studies have been conducted in this regard. The authors in [13] developed an automated diagnostic system based on multivariate linear regression for detecting knee osteoarthritis from a sample of 1024 knee X-ray images. This method achieved 82.98% accuracy in the classification of osteoarthritis. In [14], the authors developed a framework based on Zernike-based texture analysis and deep learning that improves the accuracy percentage of diagnostic systems by 11%. In [15], the authors investigated the effect of MRI and patients’ data on the prediction of knee osteoarthritis based on various deep learning models. In this study, the best performance in the diagnosis of OA was achieved by the CVAE model, with an AUC score of 66.99%. The authors of [16] suggested a stacked model based on the patellar texture and clinical features of radiological images and second-level machine learning. This model achieved an AUC score of 88.9% in the classification of knee osteoarthritis. A novel diagnostic and grading tool was developed in [17] for the analysis of X-ray images of OA based on the Kellgren–Lawrence (KL) grading system. In [18], the authors developed a novel algorithm based on various machine learning techniques, such as logistic regression, KNN, decision trees, and SVM, for the early classification of knee OA from X-ray images. This algorithm has achieved a classification accuracy of 91%. In [19], the authors suggest a phono-arthrography (PAG) technique for the detection and classification of knee OA. This technique is currently in the development phase; however, initial results have shown great accuracy in the classification of knee OA. The authors in [20] reported that they had developed a convolutional neural network (CNN) for the classification of knee OA from X-ray images. In the classification of knee OA from X-ray images, this model achieved an accuracy of 90.01%, with 90% recall and 87.8% specificity. The authors in [21] developed and analyzed the effectiveness of the AlexNeT model in predicting and classifying knee OA from X-ray images. In [22], the authors proposed an image super-resolution algorithm based on an improved multiscale wide residual network model for detecting knee OA from knee MRI scans. This algorithm achieved a classification accuracy of up to 95%. The authors in [23] suggested a localization model integrated with ONNX and YOLOv2 for the prediction of knee OA from radiographic images. This integrated model achieved 0.98 mAP for the localization of OA images. In [24], the authors proposed a fuzzy ensemble feature selection method for the prediction and classification of knee OA from radiographic images. The suggested method achieved an accuracy of 73.55% in the classification of OA.

From the studies reviewed, it is evident that most of the currently used knee OA classification methods rely on the manual interpretation of radiographic images using the Kellgren–Lawrence (KL) grading approach. However, the efficiency of this approach is restricted due to time constraints and is less accurate in diagnosing OA [25]. Recent advancements in computer-aided diagnosis have shown great promise for the diagnosis of OA, despite the challenges in using single trained machine learning models. This paper aims at addressing this challenge with the help of transfer learning by utilizing a pretrained model for diagnosis purposes. Furthermore, in our knee osteoarthritis detection study, we used transfer learning to fine-tune pretrained models (VGG-16 [26] and ResNet50 [27]) to classify X-ray images as either showing signs of osteoarthritis or not. The technical novelty lies in how we adapted and optimized this approach for our specific problem, including the use of data augmentation techniques to increase the amount of training data and the incorporation of attention mechanisms to focus the model’s attention on relevant regions of the image.

## 3. Methodology

This section will explain the general methodology followed in the development of our transfer learning model. The major components for the development of an efficient model are defined as data collection and preprocessing, model generation and analysis, visualization techniques followed to confirm the accuracy of the predictions, and evaluation metrics for the evaluation of the developed model. In this research paper, we aim to address the challenge of predicting knee osteoarthritis with high accuracy from knee X-ray images. Knee osteoarthritis is a chronic joint disease that affects millions of people worldwide and is characterized by progressive damage to the knee joint. The early detection and prediction of knee osteoarthritis are critical to improving patient outcomes and enabling early interventions. However, traditional methods for diagnosing knee osteoarthritis involve subjective evaluations by radiologists, which can be time-consuming and may not always yield accurate results. Furthermore, several machine learning and deep learning techniques have recently been developed to automate detection. However, these techniques require large amounts of labeled data for training and may result in overfitting or poor generalization performance. Transfer learning involves utilizing a pretrained neural network model on a large dataset for a similar task and fine-tuning it on a smaller dataset for a specific task. By doing this, the model can leverage the learned features from the pretrained model to improve its performance on the specific task. Compared to traditional methods, the transfer learning approach suggested in this paper, offers several advantages. Firstly, transfer learning enables the model to learn from a large dataset of X-ray images and adapt to a smaller dataset of knee osteoarthritis images, resulting in improved accuracy and reduced training time. Secondly, the pretrained model has learned useful features that can be transferred to the specific task, reducing the need for extensive data processing and feature engineering.

### 3.1. Data Collection and Preprocessing

Data collection is the primary step in developing an efficient transfer learning model (TL). TL is data-dependent, and without a high-quality training dataset, high-performing models can also generate low predictive accuracy. One of the largest sources for collecting high-quality data is Kaggle. The main reason for choosing Kaggle as a data collection source is that it is a publicly available data source, which makes it easier for researchers to develop effective solutions. In this study, we also used the dataset from Kaggle, which is available at [28]. The description of the dataset is given in Table 1. 

The dataset contains 3 folders, namely, the training set, testing set, and validation set, which further contain 2 subfolders, namely, normal and osteoarthritis. The training set is used to train the transfer learning model, and the testing set contains previously unseen data that are used to test the transfer learning model. The validation set is used for fine-tuning the parameters of the training dataset. Once the dataset is collected, preprocessing is performed to transform the collected data into a format that can be best used by the transfer learning models. To ensure the quality of data in the dataset used for training our model, initially, we started by cleaning the dataset by removing data that contained inconsistencies or any missing data label values that might affect the results of our model. Furthermore, several data preprocessing techniques have been applied to enhance the quality of the images such as:

- Denoising: We applied image denoising techniques to remove noise from the X-ray images. This is important because X-ray images may have varying levels of noise due to factors such as the imaging equipment and patient movement during the scan. Noise can reduce the quality of the images, making it more difficult for the transfer learning model to accurately identify knee osteoarthritis. We applied the median filtering technique to remove noise from an X-ray image by replacing each pixel with the median value of its neighboring pixels. We applied a 2D median filter with a kernel with the size 5 × 5. This parameter determines the size of the sliding window used to compute the median value for each pixel. A larger kernel size, such as 5 × 5, provides more significant noise reduction but may also blur important details in the image compared to a smaller kernel size, such as 3 × 3. A 5 × 5 kernel size means that the filter considers a 5-pixel neighborhood around each pixel to compute the median value. The significance of applying a 2D median filter with a 5 × 5 kernel size lies in its increased ability to remove noise, such as salt-and-pepper noise, from X-ray images. However, due to the larger kernel size, there is a trade-off in terms of preserving the edges and fine details in the images. This can result in a smoother image that might lose some important features, but it is still found to be effective for transfer learning models.
- Image enhancement techniques: Image enhancement techniques were applied to improve the clarity and contrast of X-ray images. This makes it easier for the transfer learning model to identify subtle features of knee osteoarthritis, such as the narrowing of joint spaces or the presence of osteophytes. We used the histogram equalization technique, contrast stretching, sharpening filters, and Gaussian smoothing techniques to enhance the quality of images. Specifically, we used adaptive histogram equalization with the clip limit set to 2.0 and the title grid size set to 8 × 8. Adaptive histogram equalization computes the equalization locally in small regions (tiles) rather than globally for the entire image. This allows the method to adapt to local contrast variations, improving the overall image quality. The clip limit determines the maximum height of the local histogram that is used for equalization. Any histogram peaks higher than the clip limit will be “clipped”, and the excess intensity values will be redistributed uniformly among the other bins in the histogram. This helps in limiting the amplification of noise. A higher clip limit will allow more contrast enhancement, but it may also amplify noise. In our work, a clip limit of 2.0 was used, which means that the maximum height of the histogram is twice the average height. The tile grid size defines the number of rectangular regions (tiles) into which the image is divided for local equalization. Each tile is processed independently and then combined to form the final equalized image. Smaller tile sizes result in more localized equalization, better adapting to local contrast variations. However, smaller tiles may also cause visible boundaries between tiles in the final image. A larger tile size reduces this issue but may not capture local contrast variations as effectively. In our work, an 8 × 8 tile grid size was used, which means the image is divided into 64 equal-sized rectangular tiles for processing. Further, contrast stretching was performed by stretching the intensity ranges between the 1st and 99th percentiles of the original intensity range. This means that pixel values below the 1st percentile are set to the minimum intensity value (0), and the pixel values above the 99th percentile are set to the maximum intensity value (255 for an 8-bit image). This approach removes the extreme intensity values that could be caused by noise or artifacts, focusing on the more relevant intensity range that covers 98% of the pixel values in the image. It effectively redistributes the intensity values more uniformly across the available range, enhancing the contrast. After determining the 1% and 99% percentile values, the image is rescaled so that its intensity range spans from 0 to 255 (for an 8-bit image). This step ensures that the full range of intensity values is utilized, maximizing the contrast of the resulting image. For example, if the 1st percentile corresponds to an intensity value of 20 and the 99th percentile corresponds to an intensity value of 230, then the contrast stretching operation will rescale all pixel values proportionally, such that the pixel with the value of 20 becomes 0 and the pixel with the value of 230 becomes 255. To enhance the edges and fine details in an image and make the transfer learning model capable of identifying important features from an image, unsharp masking is performed. To perform unsharp masking, two parameters are defined, the radius and the amount. The radius parameter determines the size of the Gaussian blur kernel used to create the blurred image. A larger radius will result in more significant blur, which in turn will emphasize larger edges and features in the image during the unsharp masking process. In our model, we used a radius of 2, which means that the Gaussian blur kernel will consider a 2-pixel-radius neighborhood around each pixel to compute the blur. Another parameter used is “amount”. This parameter controls the degree of sharpening applied to the image. It is the scaling factor used to combine the original image with the difference between the original and blurred images. A higher amount value will result in more pronounced sharpening, while a lower value will result in a subtler effect. For our model, we used an amount value of 1.0, which means that the difference between the original and blurred images is added directly to the original image, resulting in a moderate level of sharpening. Finally, to reduce noise, a Gaussian filter is used, which is characterized by two parameters: kernel size and standard deviation. The kernel size determines the size of the filter applied to the image. A larger kernel size results in more significant blurring, while a smaller size preserves finer details. We used a kernel size of 5 × 5, which means that the filter considers a 5-pixel neighborhood around each pixel. Another parameter, called the standard deviation with sigma value 1, is used to control the spread of the Gaussian function used to compute the filter’s weights. A higher sigma value leads to more extensive blurring, while a lower value retains sharper edges. A sigma value of 1 indicates a moderate amount of blurring. Gaussian smoothing with these parameters helps reduce noise while preserving essential image features, which can improve the performance of transfer learning models.
- Transformation: In our work, image transformation techniques were applied to standardize and augment the X-ray images for better model training. Firstly, the images were resized to standard dimensions of 512 × 512 pixels, ensuring consistency across the dataset and enabling the transfer learning model to effectively process the images. The images are then divided into batches of 32, which helps optimize the training process by reducing the memory requirements and allowing the model to learn from multiple examples simultaneously. To further augment the dataset, image rotation and flipping techniques were employed. Image rotation involves rotating the X-ray images by a specific angle, such as 90, 180, or 270 degrees, generating new variations of the images that help the model generalize better to unseen data. Flipping the images, horizontally and vertically, also creates new variations, further enhancing the dataset’s diversity. These augmented images provide the transfer learning model with additional examples, enabling it to learn more robust features associated with knee osteoarthritis and improving its overall performance.
- Sampling: In this work, the sampling technique played a crucial role in selecting a representative subset of X-ray images for training the transfer learning model. Stratified sampling was employed to create a balanced dataset with respect to factors such as age, gender, and disease severity. Stratified sampling works by dividing the entire population of X-ray images into distinct groups, or strata, based on these factors. From each stratum, a proportional number of samples are randomly selected to ensure the adequate representation of all the groups in the final dataset. By using stratified sampling, the transfer learning model is trained on a diverse and representative set of examples, preventing any potential bias that could arise from an imbalanced dataset. This method ensures that the model is exposed to a broad range of cases, including different age groups, genders, and varying degrees of disease severity, which leads to more accurate and reliable predictions for different subgroups of patients. Overall, the application of stratified sampling contributes to a more robust and generalizable transfer learning model that can accurately predict knee osteoarthritis across various patient populations.
- Feature extraction: Feature extraction is carried out to identify the important features for the model to predict knee osteoarthritis. In this study, we used pretrained VGG16 [26] and ResNeT50 [27] models, which were trained on millions of natural images, to extract features from the knee X-ray images. By fine-tuning the VGG16 and ResNeT50 models on the knee X-ray images, the transfer learning model learns to identify the key features associated with knee osteoarthritis, such as joint space narrowing and osteophyte formation.

In our transfer learning models, we developed several helper functions using Python libraries, including TensorFlow, scikit-learn, NumPy, and Matplotlib, to streamline the model’s workflow and improve efficiency. These helper functions focus on specific tasks, such as loading and preprocessing images, creating confusion matrices, and predicting and plotting results. The primary purpose of the “load and prep image” function is to load an image, preprocess it, and prepare it for input into the transfer learning model. To achieve this, the function first reads the image file using TensorFlow’s tf.io.read_file function. It then decodes the image into a tensor using tf.image.decode_image. After decoding the image, the function resizes it to the desired shape with tf.image.resize, ensuring that all images have a consistent size when fed into the model. Lastly, the pixel values are scaled to range between 0 and 1 by dividing them by 255. This standardized preprocessing ensures that the images are in the appropriate format for the model. The “make confusion matrix” function is designed to compute and visualize the confusion matrix based on the predicted and true labels. It first calculates the confusion matrix using scikit-learn’s confusion_matrix function. The resulting matrix is then visualized using Matplotlib’s imshow function. The colormap, normalization, and labels can be customized according to the user’s preferences. Optionally, the function can save the confusion matrix plot as an image file. This function provides a clear visual representation of the model’s performance, helping identify any discrepancies or areas that need improvement. The “pred and plot” function is a combination of the “load and prep image” function and the model’s prediction capabilities. It starts by using the “load and prep image” function to process the input image, ensuring it is in the appropriate format for the model. Next, the function utilizes the trained model’s predict method to make a prediction on the preprocessed image. Depending on the problem, the function can handle single-class or multi-class classification. Once the prediction is made, the function plots the image using matplotlib.pyplot.imshow, with the predicted class displayed as the title. This function allows users to quickly visualize the model’s prediction on any given image, making it easier to understand the model’s performance. These helper functions provide several advantages, such as reusability, consistency, and modularity. They enable the efficient reuse of code across multiple models, simplify maintenance and debugging by breaking down complex tasks, and ensure that common tasks are performed consistently throughout the entire codebase. This approach leads to more consistent results, reduced output variance, and an overall more efficient and reliable transfer learning model.

**Figure 1:**
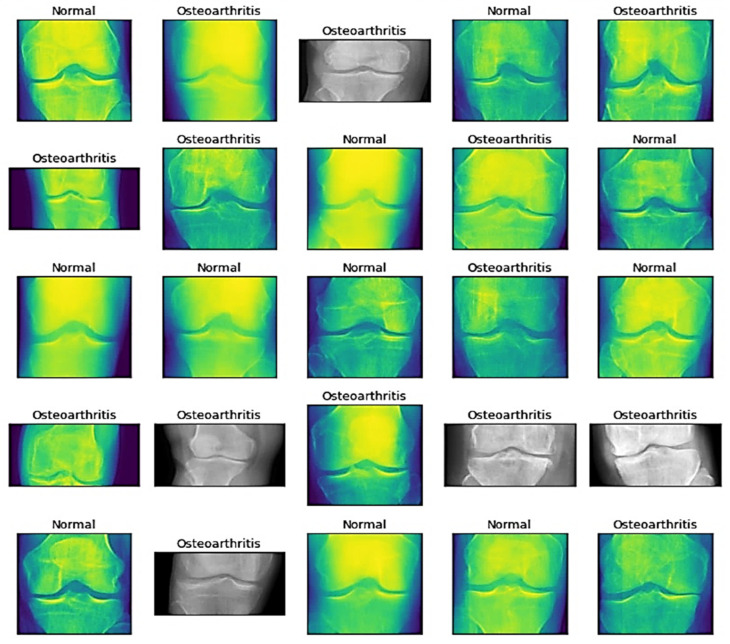
Schematic representation of the preprocessed images from the dataset.

The images obtained after preprocessing are depicted by Figure 1.

### 3.2. Model Architecture

**Figure 2:**
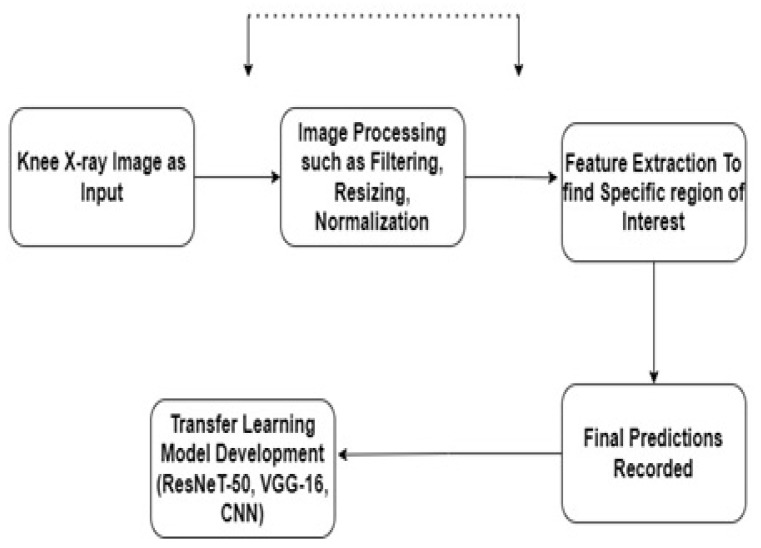
Block diagram of the proposed methodology.

In this study, we employed a transfer learning model based on a sequential convolutional neural network (CNN), Residual Network 50 (ResNeT-50), and Visual Geometry Group 16 (VGG-16) [27,28]. Initially, we trained the models individually and then passed the output to the next models for generating transfer learning models. Transfer learning (TL) is defined as a technique in which a model is trained on a task or dataset, which, by default, is the ImageNet database, and reused on a second related task for predictions. In TL, we first train a base network, which, in our case, is a sequential CNN, on a base task, and then we transfer the learned features to a second network, which will be trained on a larger dataset. The block diagram of the proposed methodology is shown in Figure 2. 

This technique will only work if the feature of the dataset is general and suitable to both the base and target models, instead of being specific to the base model [29,30]. Here, we used transfer learning to fine-tune the pretrained VGG-16 and ResNeT-50 models with the sequential CNN as the base model. In our TL model, there is a source domain (S_D_) with a learning task (L_T_) and a target domain (T_D_) with a learning task (L_L_). Let the target prediction function be defined as (T_P_(.)) in T_D_; with the help of the knowledge in S_D_ and L_T_, the TL model tries to improve the learning of the target prediction function by the technique defined by [31]. In our model, the domain is defined as a pair, such as {$\lambda ,Z\left(Y\right)$}, and this domain follows the rule S_D_ ≠ T_D_, ⇒ either $\lambda_{Source}\neq \lambda_{Target}$ or $Z_{Source}\left(Y\right)\neq Z_{Target}\left(Y\right)$. This means that, in our TL model, in between the source model (CNN) to the target model (VGG-16 or ResNeT-50), the features or distributions are different. In addition, let a task be defined as the pair {$\beta ,Z\left(M|Y\right)$} such that L*_Source_* ≠ L*_Target_* ⇒ either $\beta_{Source}\neq \beta_{Target}$ or Z (M*_Source_*|Y*_Source_*) ≠ Z (M*_Target_*|Y*_Target_*). This means that both the source and target domains are equal, along with having the same learning tasks, so this will behave as a classical machine learning problem. When we have different domains, it means that either the features between the domains are different or the marginal distributions between them are different. When the learning tasks are different, then either the label spaces between the domains are different or the conditional probability distributions are different. This can be further understood by the document classification problem as described in [32]. 

Another model used in transfer learning is a pretrained VGG-16 model. VGG-16 consists of a total of 16 layers, out of which 5 layers are pooling layers having dimensions of 112 × 112, 56 × 56, 28 × 28, 14 × 14, and 7 × 7. The pooling layers are used for extracting features from the layers in a way such that extraction from pooling layer-1 will transform the image into a grid of size 112 × 112, in which each grid has a feature vector corresponding to the activation function in the activation map [33]. The major reason for choosing VGG-16 in our work is that this model is already pretrained on the ILSVRC dataset [33], containing more than 1 million images with different categories, which makes the VGG network capable of representing many objects. 

**Figure 3:**
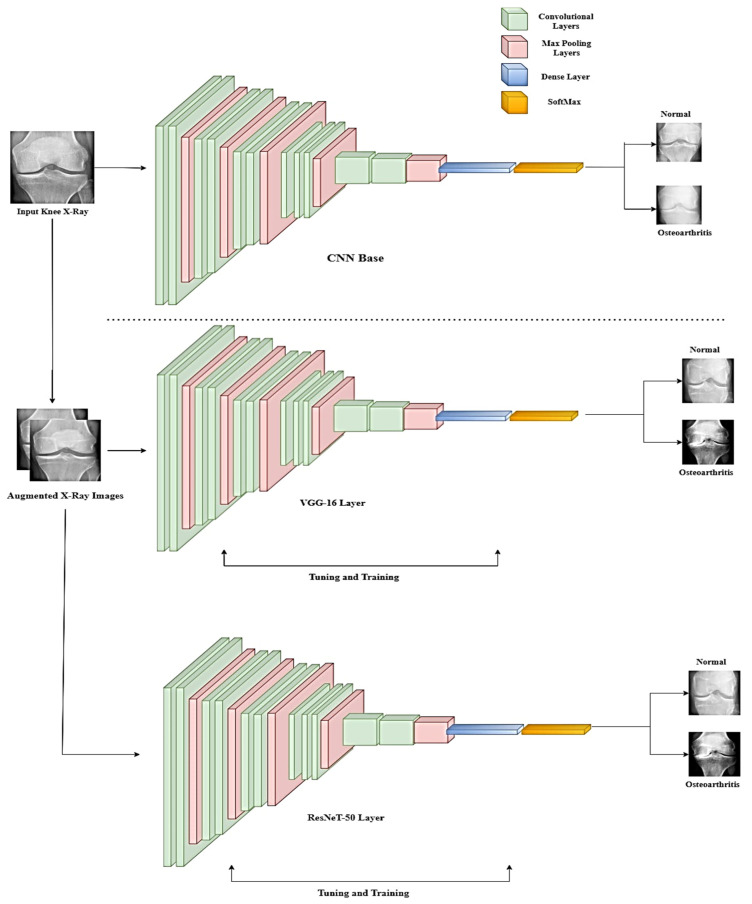
Schematic representation of the overall system architecture.

In a VGG-16 model, the process of convolution is defined as:

(1) $$Z\left(a,b\right)=\left(M*N\right)\left(a,b\right)=\sum_{l}\sum_{j}M\left(l,j\right)N\left(a-l,b-j\right)$$

where *l* and *j* are defined as the kernel’s dimensions, and (*a*, *b*) are defined as the dimensions for the matrix for which the convolution is calculated [26,34]. After the calculation of convolution, each convolutional layer is followed by a ReLU layer that uses the maximum pooling layers for sampling purposes. Consider a dataset having *S* samples as *Q*(1), *R*(1), …, *Q*(*S*), with *R*(*S*) used to train the model. Then, the cost function for the model is calculated as [26]:

(2) $$\lambda \left(\phi ,\alpha \right)=\left[\frac{1}{S}\sum_{i=1}^{s}\left(\frac{1}{2}{\left\|\left.M_{\phi ,\alpha }\left(Q^{i}-R^{i}\right)\right.\right\|}^{2}\right)\right]+\frac{Y}{2}\sum_{k=1}^{f_{k-1}}\sum_{i=1}^{h_{k}}\sum_{j=1}^{h_{k+1}}{\left(\phi_{ij}^{\left(k\right)}\right)}^{2}$$

where $M_{\phi ,\alpha }$ is defined as the neural network model, $\phi_{ij}^{\left(k\right)}$ is defined as the connection weights between the layers, and α is the bias term of the hidden layers of the neuron. This equation helps in reducing weights, which further helps in preventing the overfitting of the model. The overall architecture of the TL model is presented in Figure 3.

After VGG-16, the pretrained ResNeT-50 model is used. The ResNeT network belongs to a family of residual networks that are 50 layers deep. A typical ResNeT-50 architecture consists of 5 convolutional blocks, including smaller convolutional blocks of 1, 3, 4, 6, and 3 blocks, respectively. These building blocks are defined as:

(3) $$Z=T\left(y,F_{j}\right)+y$$

where *y* and *Z* represents the input and output vectors of the layers, and the function *T* is defined as the learning ability of the model during training [35]. The input to the model is given as an image of size 224 × 224, and the first block of the model will render an output of 64 feature maps with the size 112 × 112 pixels. As the convolution progresses, the number of features increases with the depth of the network. In the end, the network extracts 2048 features with a size of 7 × 7 pixels. Finally, the classification is performed by the average pooling layer and fully connected layers with a SoftMax function, which is defined as [35]:

(4) $$\phi \left(\lambda_{y}\right)=\frac{exp\left(\lambda_{y}\right)}{\sum_{p}exp\left(\lambda_{p}\right)}$$

We selected VGG16 and ResNet50 as the pretrained models for detecting knee osteoarthritis from X-ray images using transfer learning because of their proven effectiveness in similar medical image analysis tasks and their complementary strengths. VGG16 has shown impressive results in various medical image classification tasks, including skin lesion classification and lung nodule detection [36]. Its ability to learn meaningful features from medical images made it a suitable choice for our study on knee osteoarthritis detection. Additionally, VGG16’s relatively simple architecture allows for easier interpretation and customization, which can be advantageous when adapting the model for domain-specific tasks such as osteoarthritis detection. ResNet50, on the other hand, has demonstrated exceptional performance in a wide range of image classification tasks, including those involving medical images. The residual connections in ResNet50 enable the model to learn more complex features, which can be beneficial when dealing with subtle variations and patterns present in medical images such as X-rays. This ability to learn intricate features allows ResNet50 to potentially capture the nuances required for accurate knee osteoarthritis detection. By employing both VGG16 and ResNet50, we aimed to leverage their complementary strengths: VGG16’s simple architecture for easier customization and ResNet50’s ability to learn complex features for capturing subtle patterns in X-ray images. This combination increased the likelihood of achieving better performance in knee osteoarthritis detection compared to using a single pretrained model or other models that might not have these specific advantages.

While there are other pretrained models available, such as InceptionV3 [37] or EfficientNet [38], we focused on VGG16 and ResNet50 due to their successful track records in medical image analysis tasks and the unique characteristics that make them well-suited for detecting knee osteoarthritis from X-ray images using transfer learning. 

For our transfer learning model using VGG16 and ResNet50, we experimented with different hyperparameter configurations to optimize performance. The key hyperparameters we focused on were the learning rate, batch size, and the number of epochs. We tested various learning rates, such as 0.001, 0.0001, and 0.00001, to determine the most suitable value. After evaluating the model’s performance, we chose a learning rate of 0.00001, as it has been proven to work well in practice for fine-tuning pretrained models. In addition to the learning rate, we also experimented with various batch sizes, such as 16, 32, and 64, to find the optimal balance between the training speed and performance. After testing these values, we selected a batch size of 32, as it provided the right balance between the efficient use of computational resources and good model generalization. Furthermore, we tested different numbers of epochs, such as 20, 30, and 50, to find the best trade-off between model performance and overfitting. After evaluating the results, we chose 30 epochs, as this value provided good results while minimizing overfitting.

To fine-tune these hyperparameters, we followed a systematic approach, namely, grid search or random search. In grid search, we tested all possible combinations of hyperparameter values within a predefined range. For example, we trained the model with a learning rate of 0.00001, a batch size of 16, and 20 epochs, and then moved on to the next combination (e.g., 0.00001 learning rate, 16 batch size, and 30 epochs) until all possible combinations had been evaluated. In random search, we randomly selected hyperparameter values within a specified range and trained the model with these values. We repeated this process for a fixed number of iterations until a stopping criterion was met. By evaluating the performance of the model with different configurations, we selected the best-performing combination that balanced performance and efficiency for detecting knee osteoarthritis from X-ray images using transfer learning with VGG16 and ResNet50.

In our study, we chose specific hyperparameters for fine-tuning the pretrained VGG16 and ResNet50 models. The selection of these hyperparameters was based on their ability to balance performance and efficiency, as well as their demonstrated success in similar tasks. For the learning rate, we chose a value of 0.00001. This learning rate is smaller than the default values often used, such as 0.001 or 0.01. The smaller learning rate was selected because it allows for more gradual and stable updates of the model weights during fine-tuning. A higher learning rate, such as 0.01, might cause the model to overshoot the optimal weights and fail to converge, while a lower learning rate, such as 0.000001, could result in an excessively slow training process. The chosen learning rate of 0.00001 provides a balance between stability and training speed, making it suitable for fine-tuning pretrained models for knee osteoarthritis detection. Regarding the batch size, we opted for a value of 32. A smaller batch size, such as 8 or 16, might lead to slower convergence, as the model’s weight updates would be based on fewer samples. Conversely, a larger batch size, such as 64 or 128, might negatively impact the model’s ability to generalize to new data, as the weight updates would be averaged over a larger number of samples, potentially smoothing out important patterns. By choosing a batch size of 32, we aimed to strike a balance between the speed of convergence and the model’s ability to generalize well to new data in the context of knee osteoarthritis detection. 

As for the number of epochs, we decided on 30 epochs for our training process. A smaller number of epochs, such as 10 or 15, might not provide the models with enough exposure to the data for them to learn the relevant features for knee osteoarthritis detection, leading to underfitting. On the other hand, a higher number of epochs, such as 50 or 100, could result in overfitting, where the model becomes too specialized in recognizing the training data and loses its ability to generalize to unseen data. By training the models for 30 epochs, we aimed to minimize the risk of overfitting while ensuring that the models were sufficiently exposed to the training data to learn the relevant features for accurate knee osteoarthritis detection.

Finally, we employed the Adam optimizer for fine-tuning the models. The Adam optimizer was chosen over other optimization algorithms, such as SGD (Stochastic Gradient Descent) or RMSprop [39,40,41], due to its ability to adapt the learning rate for each parameter individually and effectively handle sparse gradients. These characteristics make the Adam optimizer particularly suitable for fine-tuning pretrained models in transfer learning settings, as it can efficiently and adaptively update the model weights.

### 3.3. Visualization Method

Visualization is important for any study, as it provides a much deeper analysis of the output obtained. This also helps in determining relationships between real-world entities and confirming the accuracy of the developed model on the dataset. For this paper, we have used the Grad-CAM visualization [42] technique. This visualization technique was used because it provides a better understanding of any prediction failures and helps in the identification of any bias while outperforming previous benchmarks. This technique is also effective in the analysis of the backpropagation algorithm, where the downsampled relevance maps are upsampled to generate a relevance heatmap. Studies such as [42] have shown that the Grad-CAM technique required no retraining and can be applied to many CNN architectures. Furthermore, the Grad-CAM technique is class-specific and can produce separate visualizations for each class present in the images. 

To generate Grad-CAM, we need to first define the gradient with respect to the feature maps of the convolution layers, which is defined by [43]:

(5) $$Q_{p}=\sum_{R}S_{R}^{p}\frac{1}{X}\sum_{k}\sum_{L}Y_{KL}^{R}$$

where $Q_{p}$ is defined as the global average pooling transformation score, and *Y* is defined as the feature map. After the calculation gradient is complete, global average pooling is performed to generate weights, which are defined as [43]:

(6) $$\beta_{R}^{p}=\frac{1}{X}\sum_{k}\sum_{L}\frac{\partial Qp}{\partial Y_{KL}^{R}}$$

where $\beta_{R}^{p}$ is defined as the weights generated, and $\frac{\partial Qp}{\partial Y_{KL}^{R}}$ is defined as the convolutional layer.

## 4. Results and Discussion

Initially, we started with our base sequential CNN model. Before loading the X-ray image training data into the model, data augmentation was carried out. As our models are data-dependent, high-quality images must be passed through the model to achieve good predictive accuracy. Data augmentation helps in improving the predictive accuracy of the model by adding more training data to the transfer learning model, thereby preventing data scarcity for the models. Data augmentation also helps in reducing the overfitting of the data and increasing the generalization ability of the models, which further improves the issues related to the class imbalance. The data augmentation performed is summarized in Table 2.

**Figure 4:**
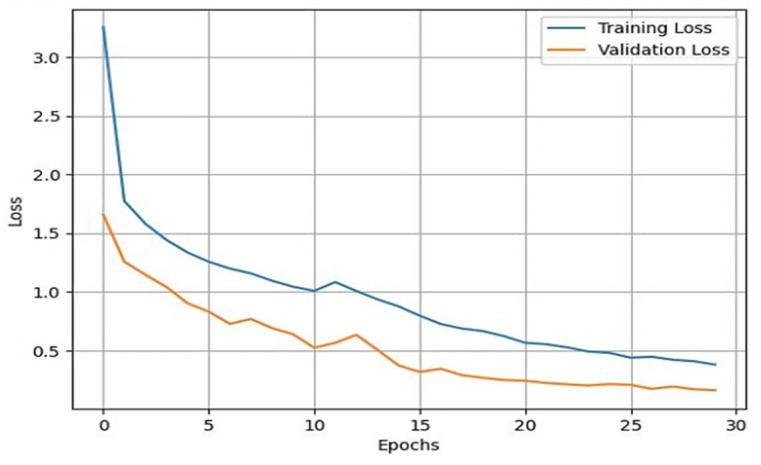
Line plot for training loss vs. validation loss.

**Figure 5:**
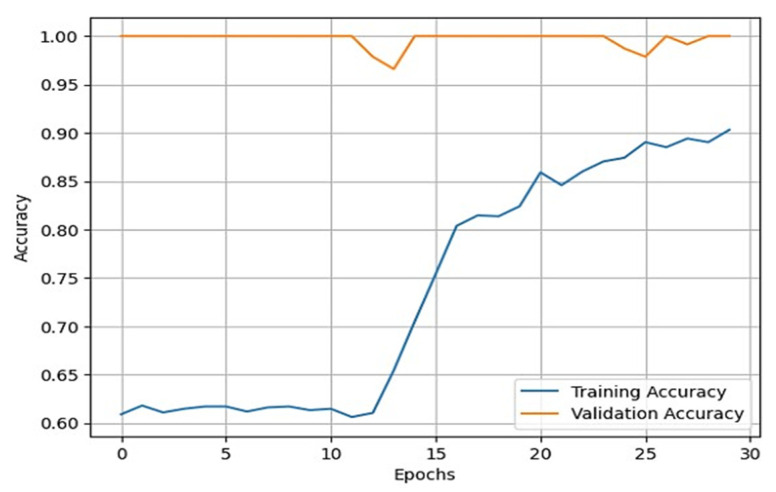
Line plot for training accuracy vs. validation accuracy.

Once the data augmentation is performed, the X-ray images are passed through the sequential CNN base model. This model is trained with the default ImageNet weights for 30 epochs of batch size 32. The validation split was set to 0.10, and the shuffle was set to True. With the developed model, we validated it through different datasets. This helped us in developing a reliable model based on its predictions on the validation data. To assess the external validity of our model, we conducted a validation process using a separate test set. We randomly split our dataset into training, validation, and test sets, with a 70-15-15 split, respectively. To provide specific details about the performance of our transfer learning model on the test set, we first need to evaluate the model using various evaluation metrics, such as accuracy, precision, recall, F1 score, and specificity. These metrics help us better understand the model’s performance in different aspects, such as correctly identifying positive cases, avoiding false positives, and balancing precision and recall. After training and fine-tuning the model using the training and validation sets, we applied the model to the test set, consisting of 845 X-ray images. By evaluating the model’s performance on this unseen data, we can assess its generalizability and ability to perform well on new data. We then compared the results obtained from our model on the test set with the performance metrics obtained from other studies to understand how our approach fares relative to existing methods. We also used the training and validation sets for model training and hyperparameter tuning and reserved the test set for the final model evaluation. During the testing phase, we used the trained model to predict the knee osteoarthritis status from the X-ray images in the test set. During the training of the model, accuracy and loss can fluctuate depending upon the cases. Generally, it is seen that if the number of epochs is increased, the accuracy increases, and the loss decreases. However, in cases of validation loss and validation accuracy, a few cases must be kept in mind. That is, if the validation loss starts increasing and the validation accuracy starts decreasing, then this indicates that our model is not learning. If the validation loss starts increasing, then validation accuracy also increases. This can cause model overfitting. If the validation loss decreases, then validation accuracy increases. In our model, we used the loss as binary cross-entropy. This shows that the developed model is learning properly and is working accurately. The validation loss and accuracy are summarized in Figure 4 and Figure 5, respectively.

From these plots, it is evident that they belong to the third case, which shows that our model is learning effectively. Additionally, our validation process showed that our model’s performance is not limited to the training and validation sets and can generalize well to new and unseen data. To generate good predictive results, we need to refine the model. In this process, we used the Adam optimizer. The Adam optimizer is an integration of the RMSP algorithm and gradient descent with the momentum algorithm [39,40,41]. The Adam optimizer requires less memory and is efficient in dealing with larger datasets. This optimizer helps us in handling sparse gradients on a noisy dataset, which generates effective predictive results. The operation of the Adam optimizer is defined by Algorithm 1 [39,40,41] as:
**Algorithm 1:** Adam Algorithm1. Initialize, $p_{0}=0$ and q_1_ 2. for j = 1, …, J do 3.   $p_{j}=\lambda_{\left(1,j\right)}p_{j-1}+\left(1-\lambda_{\left(1,j\right)}\right)z_{j}$ 4.   $\phi_{j}=\beta_{j}\left(z_{1},z_{2},z_{3},\ldots ,z_{j}\right)$ 5.   $b_{j+1}=b_{j}-\frac{\delta_{j}p_{j}}{\sqrt{\phi_{j}}}$ 6.  end

In this algorithm, b is the optimization variable, $z_{j}$ is the stochastic gradient at step j, $\lambda_{\left(1,j\right)}$ is the non-increasing sequence, and $\beta_{j}$ is an arbitrary function that generates an output vector of the same dimension as that of b. During the training of our model, we used a dropout of 0.5, which implies that randomly selected neurons are removed from the training of our model to prevent it from overfitting. The process of the removal of neurons is explained by [44]. Upon training the model, we achieved a training accuracy of 93.53% and a testing accuracy of 90.95%. 

In our study, we employed various evaluation metrics, such as precision, recall, F1 score, and specificity, to assess the performance of our transfer learning model in detecting knee osteoarthritis from X-ray images. These metrics help us understand the model’s capabilities in different aspects of classification tasks. Precision measures the proportion of true positive predictions out of all positive predictions made by the model. A high precision score indicates that the model makes fewer false positive predictions, which is crucial in medical diagnosis to avoid unnecessary treatments. Precision can be calculated as:

$$Precision\;(P)=\frac{Q}{\left(Q+R\right)}$$

where *Q* = true positive (TP) and *R* = false positive (FP).

Recall, also known as sensitivity, evaluates the model’s ability to correctly identify positive cases within the dataset. A high recall score demonstrates that the model can accurately detect most positive samples. Recall can be calculated as:

$$Recall\;(R)=\frac{P}{\left(S+T\right)}$$

where *S* = true positive (TP) and *T* = false negative (FN).

Specificity is an important metric that gauges the model’s capacity to correctly identify negative cases, or true negatives (TN). In the context of knee osteoarthritis detection, high specificity ensures that the model can accurately identify images without signs of osteoarthritis, which helps prevent unnecessary medical interventions or follow-up examinations. Specificity can be calculated as:

$$Specificity=\frac{TN}{\left(TN+FP\right)}$$

The F1 score, on the other hand, is the harmonic mean of precision and recall. It provides a balanced measure that accounts for both precision and recall, offering a more comprehensive evaluation of the model’s performance. A high F1 score suggests a good balance between precision and recall and can be calculated as:

$$F-1\;Score=\frac{\left(2*P*R\right)}{\left(P+R\right)}$$

By utilizing these evaluation metrics, we gain a better understanding of our transfer learning model’s performance in various aspects of detecting knee osteoarthritis from X-ray images and can make more informed decisions about its effectiveness and areas for improvement. The values obtained from these metrics are summarized in Table 3.

The macro average and weighted average are two methods used to calculate the performance metrics of a classification model, such as precision, recall, and F1 score. These methods are particularly relevant in multi-class classification problems or when the class distribution is imbalanced. The macro average calculates the performance metric independently for each class and then takes the average of those values. This method treats all classes equally, regardless of their size or distribution in the dataset. The macro average is particularly useful when we want to evaluate the performance of a model on minority classes, as it gives equal weight to each class. To compute the macro average for a specific metric (e.g., precision), we calculate the precision for each class individually and then take the average of those values. On the other hand, the weighted average calculates the performance metric for each class, but instead of taking a simple average, it weighs the metric for each class by the number of samples in that class. This method takes the class distribution into account, and the performance metrics for larger classes will have a greater influence on the overall average. The weighted average is useful when we want to evaluate the performance of a model while considering the class imbalance in the dataset. To compute the weighted average for a specific metric (e.g., precision), we calculate the precision for each class individually, multiply each precision value by the number of samples in that class, sum these weighted values, and then divide the sum by the total number of samples.

**Figure 6:**
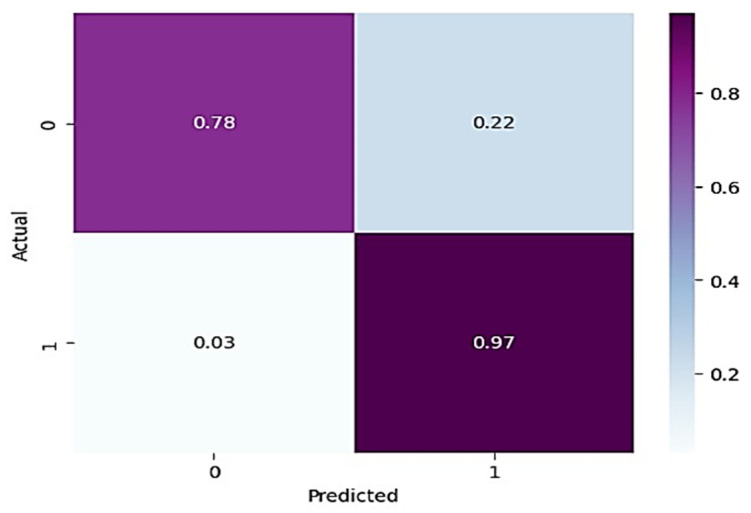
Normalized confusion matrix for base model.

In Table 3, for normal knee X-ray prediction, where osteoarthritis is absent, the model has a precision of 0.94, which means that out of all the positive predictions made by the model, 94% of them are actually true positives. The recall value of 0.78 means that the model correctly identified 78% of all actual positive samples. The F1 score of 0.85 is the weighted average of precision and recall and provides an overall measure of the model’s performance. An F1 score closer to 1 indicates a better performance of the model. Based on these results, the model has a high precision value, indicating that it has a low false-positive rate. However, the recall value is lower than the precision value, indicating that the model may have missed some positive samples. The F1 score of 0.85 indicates that the model’s performance is reasonably good, but there is still some room for improvement. On the other hand, in the presence of knee osteoarthritis in X-rays, a precision of 0.90, a recall of 0.97, and an F1 score of 0.94 mean that the model performs very well in identifying arthritis cases from knee X-ray images. A precision of 0.90 means that out of all the predicted arthritis cases, 90% of them are true arthritis cases, and the remaining 10% are false positives. A recall of 0.97 means that the model has correctly identified 97% of all arthritis cases present in the dataset. Additionally, an F1 score of 0.94 means that the model performs very well in both precision and recall. Therefore, based on these evaluation metrics, we can conclude that the arthritis detection model has a high accuracy in identifying arthritis cases from knee X-ray images. To further predict the quality of the model on the data, a normalized confusion matrix was drawn, which is shown in Figure 6. All these metrics signify that this model performs well in predicting osteoarthritis from knee X-ray images. 

Another model used for a better analysis of the results is VGG-16 [26]. This model is pretrained on the ImageNet dataset with ImageNet weights [26]. This model takes the augmented X-ray images as input, with the same Adam optimizer and other parameters as explained for the base model. This model was also trained for 30 epochs with a batch size of 32, where the learning rate of the Adam optimizer is 0.00001. The outcomes generated from training the model are recorded in Table 4.

**Figure 7:**
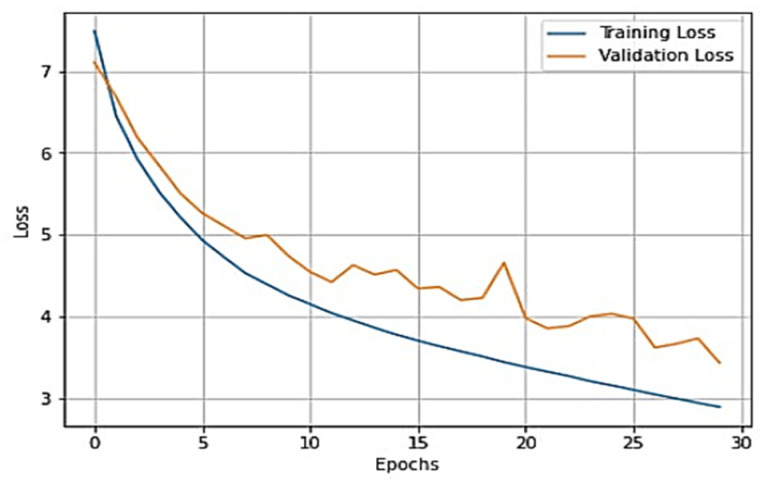
Training loss vs. validation loss plot for VGG-16.

**Figure 8:**
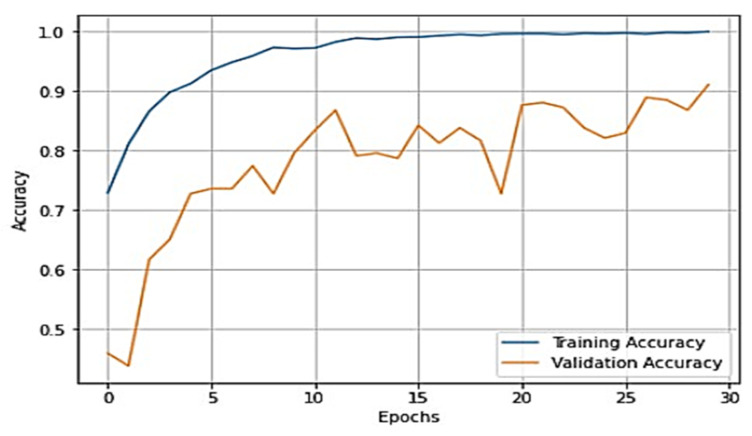
Training accuracy vs. validation accuracy plot for VGG-16.

To gain a clear understanding of the performance of the model, plots of validation loss and accuracy were obtained and are depicted in Figure 7 and Figure 8, respectively.

Upon analysis, we found that this model outperformed the previous model and achieved a training accuracy of 99.11% and a testing accuracy of 92.17%. For further investigation of the results, several performance evaluation metrics, namely, F-1 score, precision, recall, and specificity, were used. The values obtained for these metrics are recorded in Table 5.

In Table 5, the precision of the model for normal X-rays is 0.99, which means that out of all the images that the model classified as normal X-rays, 99% of them were actually normal. This indicates that the model is very accurate in identifying normal X-rays and has a low false-positive rate. The recall of the model for normal X-rays is 0.77, which means that out of all the actually normal X-rays, the model correctly identified 77% of them. This indicates that the model may have missed some normal X-rays and has a moderate false-negative rate. The F1 score for normal X-rays is 0.87, which is the harmonic mean of precision and recall. It combines both precision and recall into a single metric and provides an overall measure of the model’s accuracy. A higher F1 score indicates the better overall performance of the model. Overall, the model has a very high precision for normal X-rays, indicating a low false-positive rate. However, the recall is moderate, indicating that the model may have missed some normal X-rays. The F1 score is good, indicating an overall good performance of the model for normal X-rays. For osteoarthritis detection, the precision of 0.90 means that out of all the positive predictions made by the model, 90% were actually true positives. In the context of detecting knee osteoarthritis, this means that 90% of the X-rays that were predicted as showing signs of osteoarthritis actually have the disease. The recall of 1.00 means that out of all the actual positive cases of osteoarthritis, 100% were correctly identified by the model. In other words, the model did not miss any cases of osteoarthritis. The F1 score of 0.94 is the weighted average of precision and recall and provides a measure of the model’s overall accuracy. This score indicates that the model has a high level of accuracy in identifying cases of knee osteoarthritis from X-rays. Overall, these evaluation metrics indicate that the model has a high level of precision and recall in detecting knee osteoarthritis from X-rays, with a high overall accuracy as measured by the F1 score. This suggests that the model can be useful in assisting medical professionals in the diagnosis of knee osteoarthritis. 

**Figure 9:**
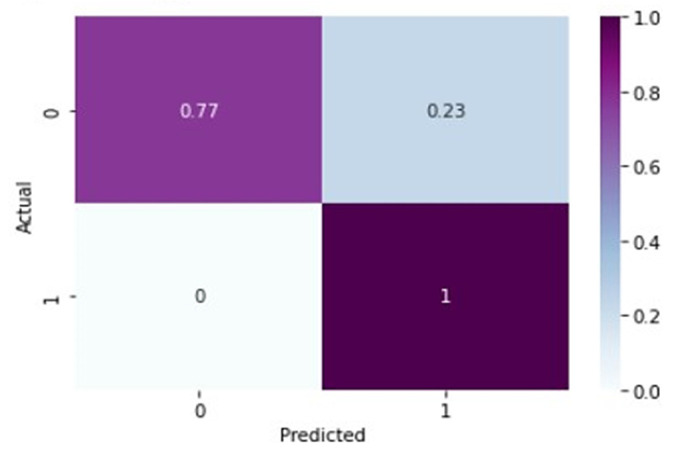
Normalized confusion matrix for VGG-16 model.

From Figure 7 and Figure 8, it is evident that when the validation loss decreases, the validation accuracy increases. This indicates that our model is learning correctly. To further predict the quality of the model on the data, a normalized confusion matrix was drawn, which is shown in Figure 9. All these metrics signify that this model performs well in predicting osteoarthritis from knee X-ray images. 

The final pretrained model used is ResNeT-50. The network architecture is very large due to an increasing number of layers. To avoid resource crashing, the ResNeT architecture is defined by the third layer in Figure 3. A typical ResNeT architecture is 50 layers deep. For more understanding of the ResNeT architecture, refer to the study in [27]. 

This model was also trained for 30 epochs by keeping the optimizer and all other parameters the same. The outcomes generated by the model are recorded in Table 6.

**Figure 10:**
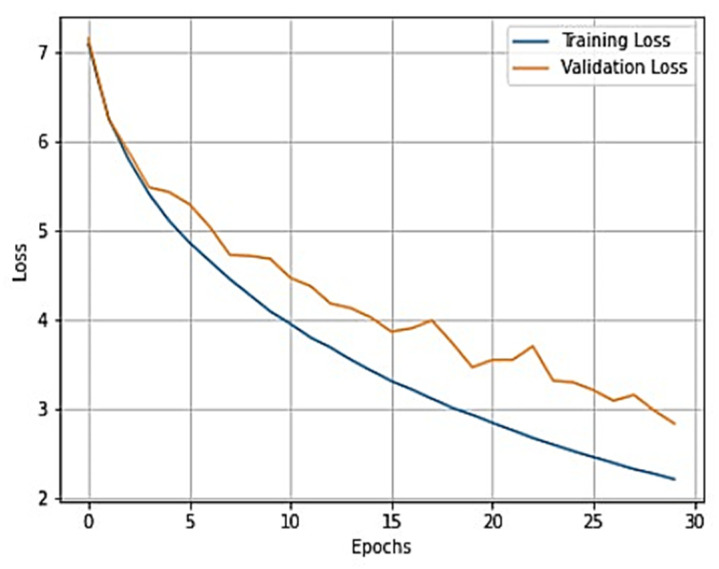
Training loss vs. validation loss plot for ResNeT-50 model.

**Figure 11:**
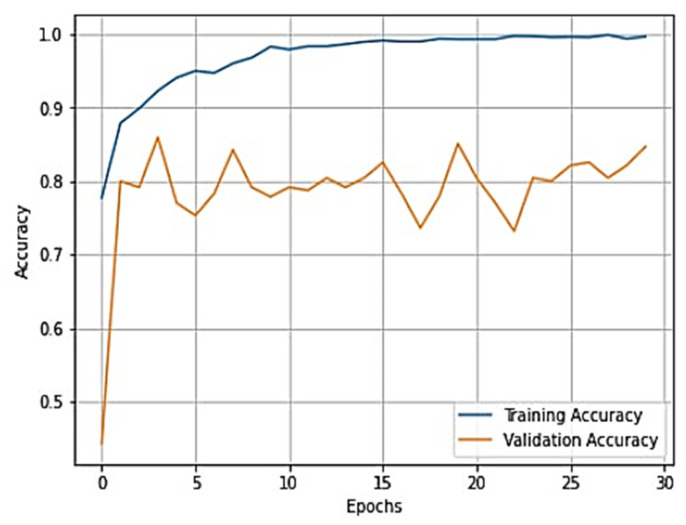
Training accuracy vs. validation loss plot for ResNeT-50 model.

The overall training accuracy achieved for the ResNeT-50 model is 98.47%, and the overall testing accuracy achieved is 90.63%. For a clear understanding of the performance of the model, plots of validation loss and accuracy were obtained and are depicted in Figure 10 and Figure 11, respectively. 

For further investigation of the results, several performance evaluation metrics, that is, F-1 score, precision, recall, and specificity, were used. The values obtained for these metrics are recorded in Table 7.

In Table 7, for normal X-rays, the precision of 0.98 indicates that out of all the predicted normal X-rays, 98% were actually normal. The recall value of 0.74 means that out of all the actually normal X-rays, only 74% were correctly identified as normal by the model. The F-1 score of 0.84 is the harmonic mean of precision and recall and gives an overall measure of the model’s performance in identifying normal X-rays. A precision score of 0.98 is relatively high, indicating that the model is good at predicting normal X-rays. However, a recall score of 0.74 indicates that the model is missing a significant number of normal X-rays, which could lead to false positives for arthritis detection. The F1 score of 0.84 indicates that the overall performance of the model is good, but there is still room for improvement, particularly in increasing the recall value. The given precision value of 0.88 for arthritis detection from X-rays suggests that out of all the predicted cases of knee osteoarthritis, 88% are actually true positive cases of knee arthritis. The recall value of 0.99 indicates that out of all the actually positive cases of knee arthritis, 99% of them were correctly identified by the model. The F-1 score of 0.93 indicates a good balance between precision and recall. Overall, these evaluation metrics suggest that the model performs well in detecting knee arthritis from X-rays with high accuracy and high recall. 

**Figure 12:**
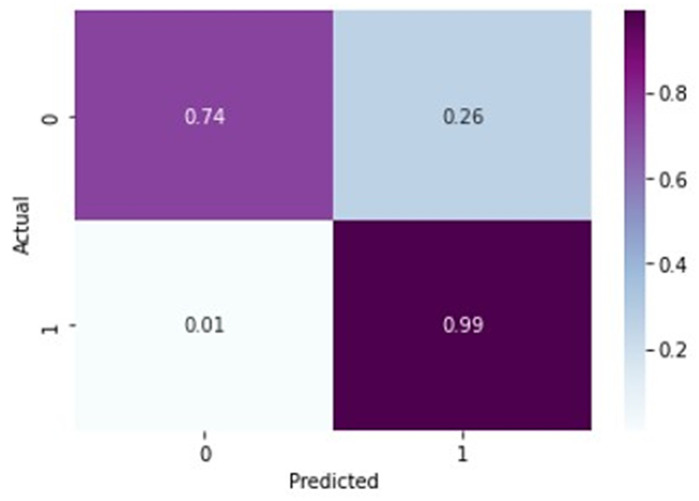
Normalized confusion matrix for ResNeT-50 model.

From Figure 10 and Figure 11, it is evident that when the validation loss decreases, the then validation accuracy increases. This indicates that our model is learning correctly. To further predict the quality of the model on the data, a normalized confusion matrix was drawn, which is shown in Figure 12. Furthermore, the overall computational time for 3836 X-ray images with the pretrained sequential CNN model is approximately 30 min; for the pretrained VGG-16 model, the overall computational time is approximately 12.8 min; and for the ResNeT-50 model, the computational time is approximately 11.51 min. In the case of knee osteoarthritis detection using X-ray images, the computational time for the three pretrained models (pretrained sequential CNN, VGG-16, and ResNet-50) provides valuable information about the feasibility and practicality of using these models in real-world settings. The pretrained ResNet-50 model has the shortest computational time among the three models evaluated, which makes it a suitable option for scenarios in which quick results are required. However, this model may not be the best option if accuracy is the most critical factor since it has the lowest reported accuracy among the three models. On the other hand, the pretrained VGG-16 model has an intermediate computational time, which makes it a suitable option for scenarios in which both accuracy and computational time are important factors to consider. Lastly, the pretrained sequential CNN model has the longest computational time among the three models. However, this model has the highest reported accuracy, which makes it the most suitable option if accuracy is the most critical factor and if the longer computational time can be accommodated. 

A comparative analysis of the accuracy percentages between various models is presented in Table 8.

**Figure 13:**
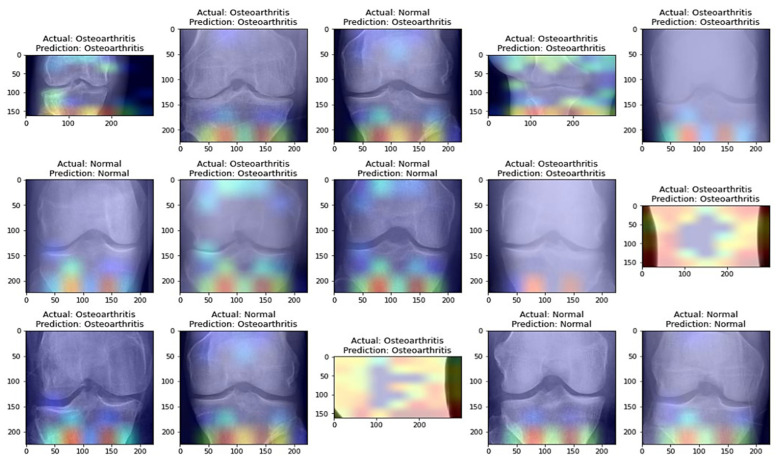
Predictions obtained with the most accurate model on randomly selected X-ray images from the dataset.

**Figure 14:**
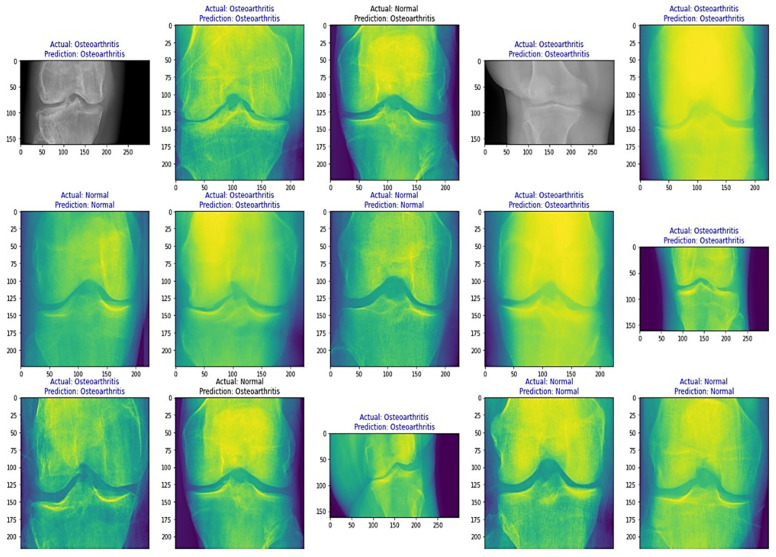
Grad-CAM visualizations of the predictions.

From Table 8, it is evident that the pretrained VGG-16 model achieved a higher training accuracy and testing accuracy and outperformed the other two models. Thus, VGG-16 is a more suitable model for the early prediction of knee osteoarthritis from X-ray images. To visualize the predictions, we generated visualizations of the predictions for some random images from the dataset to show the effectiveness of the most accurate model. The accuracy is further confirmed through Grad-CAM visualization. These are shown in Figure 13 and Figure 14, respectively.

The suggested methodology in this paper presents several advantages over other methods, including improved detection performance, reduced model training time, the efficient use of data, and the high transferability of knowledge. However, it has some disadvantages, such as model overfitting and the limited adaptability of data, which can affect the system’s performance. A comparative analysis between different advantages and disadvantages of the methodology suggested in this paper with other closely related studies is summarized in Table 9. Furthermore, a comparison of the methodology suggested in this paper was also made based on the accuracy percentage with other closely related studies and is summarized in Table 10.

In Table 9 and Table 10, the presented comparative analysis shows the performance of different models on the task of detecting knee osteoarthritis from X-rays. The accuracy percentage metric is used to compare the performance of the models. Accuracy is a measure of how well the model predicts the correct class for all the samples in the dataset. Based on the comparison, the model developed in this study achieved the highest accuracy of 92.17% compared to the other studies. The accuracies of the other studies range from 61% to 97.7%, with an average of 86.88%. This comparative analysis suggests that the model developed in this study performs better than most of the other models developed for the same task. It also suggests that detecting knee osteoarthritis from X-rays is a challenging task, as the accuracies of the other studies vary widely. The differences in accuracy could be due to various factors, such as the size and quality of the dataset used, the choice of model architecture, hyperparameters, the optimization algorithm used, and the preprocessing techniques applied to the data. The accuracy of other studies ranged from 61% to 97.7%. The studies that achieved an accuracy below 80% can be considered poor or inadequate. The studies that achieved an accuracy between 80% and 90% can be considered fair or average, while the studies that achieved an accuracy above 90% can be considered excellent. Therefore, the results of this comparative analysis suggest that the model developed in this study could be a valuable tool for detecting knee osteoarthritis from X-rays, and it outperforms most of the other models developed for the same task.

Our transfer learning approach has shown promise in detecting knee osteoarthritis from X-ray images when comparing our model’s accuracy with other studies. Nonetheless, there are certain limitations and potential issues that warrant further discussion and specificity. First, when comparing our results with other studies, it is important to consider not only the differences in datasets and preprocessing techniques but also the specific methodologies employed by each study. For instance, other studies may have used different evaluation metrics, model architectures, or training strategies, which could impact the model’s performance. Providing a more detailed comparison between our approach and the methods used in other studies would offer a better understanding of the relative strengths and weaknesses of each approach. Second, regarding the quality and size of the dataset used for training and testing, a more in-depth analysis of the dataset’s composition could reveal potential biases or the underrepresentation of certain patient subgroups. By examining the distribution of factors such as age, gender, and disease severity within the dataset, we could gain insights into how well our model generalizes to the broader population. This would also inform potential strategies for improving the model’s performance by addressing any identified shortcomings in the dataset. Third, when discussing the choice of pretrained models for transfer learning, we could delve deeper into the specific characteristics of VGG16 and ResNet50 that make them suitable or unsuitable for the task of detecting knee osteoarthritis from X-ray images. By comparing these models with other potential options, we could identify areas where alternative pretrained models might outperform VGG16 and ResNet50. This would not only provide a clearer rationale for our choice of pretrained models but also suggest avenues for future research to explore other model architectures that could potentially yield better results. By addressing these concerns and providing a more detailed examination of the limitations and potential issues, we can better understand the true capabilities of our transfer learning approach in detecting knee osteoarthritis from X-ray images and identify areas for further improvement. 

Furthermore, when comparing our results with other studies, it is important to consider not only the differences in datasets and preprocessing techniques but also the specific methodologies employed by each study. For instance, other studies may have used different evaluation metrics, model architectures, or training strategies, which could impact the model’s performance. Providing a more detailed comparison between our approach and the methods used in other studies would offer a better understanding of the relative strengths and weaknesses of each approach. Second, regarding the quality and size of the dataset used for training and testing, a more in-depth analysis of the dataset’s composition could reveal potential biases or the underrepresentation of certain patient subgroups. By examining the distribution of factors such as age, gender, and disease severity within the dataset, we could gain insights into how well our model generalizes to the broader population. This would also inform potential strategies for improving the model’s performance by addressing any identified shortcomings in the dataset. Third, when discussing the choice of pretrained models for transfer learning, we could delve deeper into the specific characteristics of VGG16 and ResNet50 that make them suitable or unsuitable for the task of detecting knee osteoarthritis from X-ray images. By comparing these models with other potential options, such as Xception V3 or EfficientNeT, we could identify areas where alternative pretrained models might outperform VGG16 and ResNet50. This would not only provide a clearer rationale for our choice of pretrained models but also suggest avenues for future research to explore other model architectures that could potentially yield better results.

Moreover, based on the results, we found that the pretrained VGG-16 model achieved the best performance among all other models. This is because of several reasons. One reason is the architecture of the VGG16 model [26]. VGG16 has a very deep architecture, which allows it to learn complex patterns and features from images. It has 13 convolutional layers, 5 max-pooling layers, and 3 fully connected layers [26]. The use of so many layers allows VGG16 to capture both low-level and high-level features of the images. Another reason is the way in which the VGG16 model was pretrained. The VGG16 model was pretrained on a very large dataset (ImageNet) with more than one million images, which may have helped the model learn general features that are useful for various computer vision tasks [26]. The pretraining process may have helped the model avoid overfitting and generalize well to new data.

## 5. Conclusions

Knee osteoarthritis (OA) is a major cause of disability in adults. The progression of OA is generally unstoppable, and there is currently no cure for it. At present, the diagnosis of OA relies on X-ray images. However, this is a manual process that may produce inaccurate results if not implemented properly. To solve this challenge, an automated approach for the prediction of OA from X-ray images is suggested in this paper. Three models, sequential CNN, VGG-16, and ResNeT-50, are suggested for predicting OA from X-ray images. All three models achieved good accuracy, greater than 90%, but the most accurate model for the prediction of OA was VGG-16, which achieved a testing accuracy of 92.17%. From our analysis, we found that under expert supervision and a large amount of high-quality data, this automated system can perform better and generate predictions while taking the least possible time. The methodology suggested in this paper is easy to operate and is cost-effective to implement in real-world scenarios. Furthermore, the findings of our study have implications beyond the scope of knee osteoarthritis detection, as they demonstrate the potential of transfer learning models to effectively handle various medical imaging tasks. The success of our approach in detecting knee osteoarthritis from X-ray images highlights the versatility of transfer learning, which could be applied to other medical imaging modalities, such as MRI or CT scans, to detect and diagnose a wide range of diseases and conditions. In clinical practice, the proposed model could serve as an essential tool for assisting healthcare professionals in making more accurate and timely diagnoses. This can, in turn, lead to improved patient care and management, as early detection and intervention are often crucial in minimizing the impact of many medical conditions. Additionally, the use of such models can help reduce the workload of radiologists and clinicians, enabling them to focus on more complex cases or spend more time with patients. By exploring the potential of transfer learning models in various medical imaging tasks, researchers and healthcare professionals can pave the way for more advanced and reliable diagnostic tools that can revolutionize the field of medical imaging and ultimately improve patient outcomes. 

Despite the advantages offered by the technique suggested in this paper, it has some challenges that may restrict its potential in the detection of knee osteoarthritis. For example, transfer learning can result in complex models that are difficult to interpret and understand, which can reduce the ability to validate and explain the results of the automated system. Pretrained models may not be fully adaptable to the specific requirements of the target task and may require fine-tuning or additional modifications to improve their performance. Furthermore, pretrained models may contain biases and inaccuracies introduced during the training process, which can impact the performance of the automated system. Our future work will attempt to resolve these challenges and use advanced techniques such as ensemble learning and reinforcement learning.

## Figures and Tables

**Table 1:** Classification of the dataset used.

Description	Number of Images
Training set	2350 X-ray images
Testing set	845 X-ray images
Validation set	641 X-ray images
Total images	3836 X-ray images

**Table 2:** Summary of data augmentation performed during model building.

Parameter	Task Performed
Rotation_Range = 20	It rotates images by angles between 0 and 20 degrees.
Width_Shift_Range = 0.01	It shifts the image along the X-axis by the input value.
Height_Shift_Range = 0.01	It vertically shifts the image by the input value.
Horizontal_Flip = False	It stops the horizontal flipping of rows and columns.
Vertical_Flip = False	It stops the vertical flipping of rows and columns.

**Table 3:** Evaluation metrics for the base model.

	Precision	Recall	F-1 Score	Support
Normal (0)	0.94	0.78	0.85	210
Osteoarthritis (1)	0.90	0.97	0.94	431
Macro Average	0.92	0.88	0.89	641
Weighted Average	0.91	0.91	0.91	641
Specificity = 0.97				

**Table 4:** Training results obtained from VGG-16.

Epochs	Loss	Accuracy	Validation Loss	Validation Accuracy
1/30	7.4912	0.7291	7.1074	0.4596
2/30	6.4551	0.8109	6.6875	0.4383
3/30	5.9190	0.8662	6.1840	0.6170
4/30	5.5223	0.8983	5.8450	0.6511
5/30	5.2077	0.9125	5.5006	0.7277
6/30	4.9329	0.9352	5.2639	0.7362
7/30	4.7257	0.9485	5.1072	0.7362
8/30	4.5274	0.9593	4.9531	0.7745
9/30	4.3915	0.9735	4.9955	0.7277
10/30	4.2561	0.9716	4.7379	0.7957
11/30	4.1487	0.9726	4.5431	0.8340
12/30	4.0387	0.9825	4.4185	0.8681
13/30	3.9491	0.9891	4.6264	0.7915
14/30	3.8583	0.9872	4.5093	0.7957
15/30	3.7755	0.9905	4.5681	0.7872
16/30	3.7012	0.9910	4.3396	0.8426
17/30	3.6342	0.9934	4.3579	0.8128
18/30	3.5685	0.9953	4.1970	0.8383
19/30	3.5088	0.9939	4.2251	0.8170
20/30	3.4371	0.9962	4.6567	0.7277
21/30	3.3787	0.9967	3.9780	0.8766
22/30	3.3227	0.9967	3.8515	0.8809
23/30	3.2670	0.9953	3.8776	0.8723
24/30	3.2060	0.9972	3.9987	0.8383
25/30	3.1530	0.9967	4.0305	0.8213
26/30	3.0979	0.9976	3.9723	0.8298
27/30	3.0439	0.9962	3.6168	0.8894
28/30	2.9908	0.9986	3.6642	0.8851
29/30	2.9383	0.9981	3.7275	0.8681
30/30	2.8906	1.0000	3.4289	0.9106

**Table 5:** Evaluation metrics for the pretrained VGG-16 model.

	Precision	Recall	F-1 Score	Support
Normal (0)	0.99	0.77	0.87	220
Osteoarthritis (1)	0.90	1.00	0.94	431
Macro Average	0.94	0.89	0.91	651
Weighted Average	0.93	0.92	0.92	651
Specificity = 0.99				

**Table 6:** Outcomes generated from training of ResNeT-50 model.

Epochs	Loss	Accuracy	Validation Loss	Validation Accuracy
1/30	7.0904	0.7773	7.1538	0.4426
2/30	6.2619	0.8790	6.2547	0.8000
3/30	5.7947	0.8988	5.8836	0.7915
4/30	5.4136	0.9229	5.4863	0.8596
5/30	5.1099	0.9409	5.4320	0.7702
6/30	4.8678	0.9499	5.2968	0.7532
7/30	4.6622	0.9470	5.0469	0.7830
8/30	4.4578	0.9603	4.7299	0.8426
9/30	4.2779	0.9678	4.7181	0.7915
10/30	4.0964	0.9830	4.6857	0.7787
11/30	3.9562	0.9792	4.4714	0.7915
12/30	3.8029	0.9835	4.3791	0.7872
13/30	3.6892	0.9835	4.1811	0.8043
14/30	3.5528	0.9863	4.1319	0.7915
15/30	3.4316	0.9896	4.0257	0.8043
16/30	3.3145	0.9915	3.8679	0.8255
17/30	3.2210	0.9901	3.9060	0.7830
18/30	3.1180	0.9901	3.9950	0.7362
19/30	3.0159	0.9939	3.7447	0.7787
20/30	2.9354	0.9934	3.4696	0.8511
21/30	2.8475	0.9934	3.5507	0.8043
22/30	2.7621	0.9934	3.5520	0.7702
23/30	2.6778	0.9976	3.7049	0.7319
24/30	2.6019	0.9972	3.3205	0.8043
25/30	2.5302	0.9957	3.3010	0.8000
26/30	2.4616	0.9962	3.2117	0.8213
27/30	2.3962	0.9957	3.0944	0.8255
28/30	2.3287	0.9991	3.1601	0.8043
29/30	2.2744	0.9939	2.9850	0.8213
30/30	2.2146	0.9967	2.8387	0.8468

**Table 7:** Evaluation metrics for the pretrained ResNeT-50 model.

	Precision	Recall	F-1 Score	Support
Normal (0)	0.98	0.74	0.84	220
Osteoarthritis (1)	0.88	0.99	0.93	431
Macro Average	0.93	0.86	0.89	651
Weighted Average	0.91	0.91	0.90	651
Specificity = 0.99				

**Table 8:** Comparative analysis of models with their accuracy.

Model	Training Accuracy (%)	Testing Accuracy (%)
Sequential CNN	93.53	90.95
Pretrained VGG-16	99.11	92.17
Pretrained ResNeT-50	98.47	90.63

**Table 9:** Comparative analysis with advantages and disadvantages of various studies.

Authors	Work Performed	Disadvantages	Advantages
[45]	Two different models, machine learning and transfer learning, were used to detect knee osteoarthritis and classify them into subtypes based on severity grading.	Not very high detection and classification accuracy. It did not perform well in multi-class labeled samples.	Achieved an accuracy of 90.8% and performed well in binary-class label classification.
[46]	A bi-directional LSTM model was used on data collected from 17 patients affected by knee osteoarthritis to estimate the flexion kinematics of joints.	This technique was not tested on a large dataset and is not very reliable for use in the real world.	This technique effectively predicts the kinematics for knee joint flexion during phases of walking in a small dataset.
[47]	A computer-assisted detection and classification technique based on multivariate information and deep learning for knee osteoarthritis is suggested.	The accuracy decreased significantly from 93.6% to 84.2%, and the reliability of this technique in the real world is questionable.	This technique not only detects knee osteoarthritis but also grades the severity of knee arthritis in various classes.
[48]	This study evaluated various deep learning techniques in detecting knee osteoarthritis.	The evaluation of the techniques is not stable, and the accuracy ranges from 54% to 93%.	Tested the efficacy of various deep learning techniques on a dataset of 2000 + knee X-ray images.
[49]	A novel technique based on Yolo-V3 to detect and classify knee osteoarthritis from X-ray images.	The technique achieved a low detection and classification accuracy of 85% and 86.7%, respectively, when evaluated on different parameters.	The suggested technique achieved satisfactory performance in detecting early stages of knee osteoarthritis.
[50]	The use of the phono-arthrography technique is suggested for detecting osteoarthritis.	The suggested technique is still under development, but initial results have shown great promise for the future diagnosis of osteoarthritis.	Initial results achieved better accuracy in the detection and classification of arthritis.
[51]	The suggested technique evaluated the various risk factors associated with knee osteoarthritis in young adults using machine learning.	The suggested technique achieved low accuracy of 78%, 56%, and 71% respectively with different input features and 67.6%, 62.3%, and 71%, respectively, with the same input features when evaluating the risk factors.	This technique not only detected knee osteoarthritis but also evaluated the risk factors associated with it.
[52]	The use of CNN was suggested for detecting knee osteoarthritis.	The suggested technique achieved an average multi-class accuracy of 61%, which is lower than that of other state-of-art techniques.	The suggested technique also performs classification of knee osteoarthritis in classes such as KL0, KL1, KL2, KL3, and KL4.
[53]	The Kellgren–Lawrence technique, along with deep learning techniques, is used to detect and classify knee osteoarthritis.	The suggested technique achieved a higher accuracy but was tested only on a dataset of 697 patients, which is a small sample.	The model achieved an overall high accuracy of 97.7% in detecting and classifying knee osteoarthritis.
This work	Transfer learning technique based on VGG-16, ResNeT-50, and CNN is suggested for detecting knee osteoarthritis from knee X-ray images.	The suggested technique was tested on a larger dataset and achieved an accuracy of 92.17%.	Although the suggested technique achieved high accuracy, it still has disadvantages such as model interpretability, bias correction, and regularization.

**Table 10:** Comparative analysis of various studies based on accuracy percentage.

References	Accuracy %
[49]	85.7% and 86% on the dataset available at [54] using the Yolo V3 model
[52]	61% on the same dataset as [54] using a CNN
[55]	86.79% and 83.57%, respectively, on a dataset curated by the authors consisting of samples from knee osteoarthritis and hip osteoarthritis patients
[56]	85.50% on the osteoarthritis severity grading dataset and osteoarthritis initiative dataset by using a deep CNN model
[57]	91.51% on the dataset available at [54] using a transfer learning CNN model.
[58]	90.06% using Yolo V2 and the CNN technique; the dataset was collected by the authors on their own, and no specifications regarding the dataset were given.
[59]	89.29% using the same dataset available at [54] and using CNN
[60]	72% using the same dataset available at [54] and using CNN
[61]	71.33% by using an automated CNN technique on the dataset curated by the authors on their own.
This Work	92.17%

## Data Availability

The data in this research paper will be shared upon request made to the corresponding author.
